# Adaptation shapes spike train correlations: theory and application to tinnitus

**DOI:** 10.1186/1471-2202-13-S1-P146

**Published:** 2012-07-16

**Authors:** Gabriel Koch Ocker, Thanos Tzounopoulos, Brent Doiron

**Affiliations:** 1 Department of Neuroscience, University of Pittsburgh, Pittsburgh, PA 15260, USA; 2 Center for the Neural Basis of Cognition, University of Pittsburgh and Carnegie Mellon University, Pittsburgh, PA 15213, USA; 3 Departments of Otolaryngology and Neurobiology, University of Pittsburgh, Pittsburgh, PA, 15261, USA; 4 Department of Mathematics, University of Pittsburgh, Pittsburgh, PA 15260, USA

## 

In many neural systems, neurons’ firing rates in response to a suprathreshold current decrease with time. Outward currents, activated by high voltage (M-current) or calcium (AHP current), are a major source of this spike-frequency adaptation (SFA) [1,2]. SFA linearizes a neuron’s firing rate sensitivity to static inputs [3], shapes the transfer of dynamic inputs [4], and introduces negative inter-spike interval correlations that can increase information transfer [5-7]. However, these results pertain to single cell transfer, and do not elucidate the effect of SFA on pairwise or higher-order interactions between neurons. Our work aims to determine how SFA affects the output spike train correlation from a pair of neurons receiving correlated input fluctuations. We motivate our theoretical work from recent experimental work in characterizing SFA in an animal model of tinnitus.

Tinnitus is characterized by the persistent perception of a high frequency subjective sound and by an increase in both firing rates and synchrony in many auditory centers [8]. Cellular hyperexcitability would explain both the increased firing rates and increased spike train synchrony of tinnitus for neurons without SFA, according to recent theory that links firing rate and pairwise correlation [9]. In a mouse model of tinnitus, the principal cells of the dorsal cochlear nucleus (DCN) have a significantly weaker adaptation current (Li & Tzounopoulos, personal communication). We adapt the results of [10] to the case of spike-driven currents and investigate how SFA affects the relation between firing rates and spike train correlations. We show how adaptation currents consistent with the DCN neuron physiology rule out simple DCN hyper-excitability as a source of the increased DCN synchrony observed in tinnitus. Rather, our theory shows how the weakened adaptation reported in neurons from animals with behavioral evidence of tinnitus both increases firing rate and spike train correlations. Our work shows that the biophysical correlates of tinnitus are consistent with the spike train correlates of tinnitus, and that this link is not obvious from past theories linking firing rates and spike train correlations because of the presence of SFA. These results reveal that the biophysical mechanisms giving rise to hyperactivity in tinnitus and other neural pathologies have specific implications for the pairwise statistics and population coding of the affected neurons.
